# Exosomes isolated from TNF‐α‐treated bone marrow mesenchymal stem cells ameliorate pelvic floor dysfunction in rats

**DOI:** 10.1111/jcmm.18451

**Published:** 2024-06-19

**Authors:** Chenchen Zhou, Yuelin Wu, Sheng Wan, Liqun Lou, Shengyi Gu, Jing Peng, Shifeng Zhao, Xiaolin Hua

**Affiliations:** ^1^ Department of Obstetrics, Shanghai First Maternity and Infant Hospital, School of Medicine Tongji University Shanghai China; ^2^ Shanghai Key Laboratory of Maternal Fetal Medicine, Shanghai First Maternity and Infant Hospital, School of Medicine Tongji University Shanghai China

**Keywords:** exosomes, pelvic floor dysfunction, stem cells, TNF‐α, treatment

## Abstract

Exosomes derived from bone marrow‐derived mesenchymal stem cells (BMSCs) can alleviate the symptoms of pelvic floor dysfunction (PFD) in rats. However, the potential therapeutical effects of exosomes derived from BMSCs treated with tumour necrosis factor (TNF)‐α on the symptoms of PFD in rats are unknown. Exosomes extracted from BMSCs treated with or without TNF‐α were applied to treat PFD rats. Our findings revealed a significant elevation in interleukin (IL)‐6 and TNF‐α, and matrix metalloproteinase‐2 (MMP2) levels in the vaginal wall tissues of patients with pelvic organ prolapse (POP) compared with the control group. Daily administration of exosomes derived from BMSCs, treated either with or without TNF‐α (referred to as Exo and TNF‐Exo), resulted in increased void volume and bladder void pressure, along with reduced peak bladder pressure and leak point pressure in PFD rats. Notably, TNF‐Exo treatment demonstrated superior efficacy in restoring void volume, bladder void pressure and the mentioned parameters compared with Exo treatment. Importantly, TNF‐Exo exhibited greater potency than Exo in restoring the levels of multiple proteins (Elastin, Collagen I, Collagen III, IL‐6, TNF‐α and MMP2) in the anterior vaginal walls of PFD rats. The application of exosomes derived from TNF‐α‐treated BMSCs holds promise as a novel therapeutic approach for treating PFD.

## 1 INTRODUCTION

Pelvic floor dysfunction (PFD) results from diverse factors that weaken the supportive tissues of the pelvic region, causing the displacement of pelvic organs. This leads to abnormal positioning and functioning of pelvic organs, particularly pelvic organ prolapse (POP) such as uterine prolapse, laxity of the vaginal anterior and posterior walls, and stress urinary incontinence.^1^, ^2^ The development of this condition is linked to the mechanical deterioration of the vaginal and pelvic floor tissues responsible for supporting pelvic organs. Currently, alterations in the collagen of the extracellular matrix are thought to contribute to the damage of pelvic support tissues, disrupting the integrity of the pelvic floor structure and giving rise to PFD.^3^, ^4^

As the global aging population continues to grow, POP has garnered attention from medical researchers worldwide due to its substantial impact on the quality of life for women, even leading to psychological burdens.^5^ In severe cases of PFD, surgical intervention is often necessary, but the effectiveness of traditional surgery is not consistently satisfactory. Despite the recognition in recent years of the anatomical restoration effects of pelvic floor reconstruction surgery, challenges persist regarding postoperative complications.^6^, ^7^, ^8^, ^9^ Hence, there is an urgent clinical need to identify a minimally invasive approach that addresses the pathogenesis of PFD and is both safe and effective for its treatment.

In our earlier investigations, we discovered that the transplantation of bone marrow mesenchymal stem cells (BMSCs), exosomes derived from BMSCs and exosomes obtained post‐transfection of BMSCs significantly enhanced PFD in rats.^10^ Compared with mesenchymal stem cells (MSCs)‐based cell therapy, exosome treatment offers advantages such as heightened efficiency, lower immunogenicity and the capability for long‐term storage at low temperatures.^11^, ^12^ While there are observed differences in content composition and levels among exosomes derived from MSCs of various origins, their basic morphological and phenotypic characteristics, along with their fundamental functions in repairing acute injuries and preventing fibrosis, demonstrate a high level of consistency.^13^, ^14^

Conversely, the total amount and content composition of exosomes secreted by the same MSCs under distinct physiological and pathological conditions exhibit notable variations, with their functions potentially conflicting.^15^ Research indicates that pretreatment with lipopolysaccharide (LPS) significantly amplifies the paracrine effects of MSCs, elevates the expression of anti‐inflammatory cytokines and encourages the activation of M2 macrophages, compared with untreated MSCs.^16^ Studies also highlight the ability of exosomes released by MSCs exposed to inflammatory cytokines (IFN‐γ and TNF‐α) to regulate macrophage polarization.^17^, ^18^

Therefore, from a clinical standpoint, this study initially compared the inflammatory status of PFD patients, subsequently stimulated BMSCs with TNF‐α, extracted exosomes and explored their therapeutic effects on PFD in rats.

## 2 MATERIALS AND METHODS

### 2.1 Patient sample collection

The participants in this research were chosen from among surgical patients in our department. Those with POP underwent either vaginal anterior wall repair or anterior pelvic floor reconstruction surgery, following the Pelvic Organ Prolapse Quantification (POP‐Q) system, as evaluated by at least two senior gynaecologists. The primary focus was on assessing the degree of uterine prolapse and varying degrees of anterior and posterior vaginal wall protrusion. The control group comprised individuals of the same age range who had undergone total hysterectomy for different gynaecological conditions during the same timeframe. Exclusion criteria for the study encompassed patients with cervical precancerous lesions, early‐stage cancer or PFD.

All patients in each group had experienced natural menopause and had not used systemic hormones in the 3 months leading up to the surgery. They had no history of neurological or respiratory system diseases, functional ovarian tumours, metabolic diseases, connective tissue diseases, prior pelvic floor surgeries, genitourinary infections, pelvic deformities or related conditions.

Patients received preoperative information about the study and provided signed informed consent forms. Surgical procedures for the POP group involved either vaginal anterior wall repair or anterior pelvic floor reconstruction, while the control group underwent either vaginal or abdominal hysterectomy. During surgery, a cold knife was utilized to obtain a full‐thickness tissue sample from the region near the vault of the vagina, measuring approximately 1.0 cm × 1.0 cm. The tissue was rinsed with physiological saline, placed in a sterile, enzyme‐free EP tube labelled for identification, promptly immersed in liquid nitrogen and stored in a − 80°C freezer within 20 min for subsequent frozen preservation.

The study was approved by Shanghai First Maternity and Infant Hospital, School of Medicine, Tongji University, and written consent was obtained from the participant.

### 2.2 Primary rat BMSC isolation and culture

The research utilized 6‐month‐old female Sprague‐Dawley rats and received approval from the ethics committee at Shanghai First Maternity and Infant Hospital. The femurs of the rats were dislocated under anaesthesia, and bone marrow cells were flushed using 10 mL syringes with 5–10 mL cold Iscove's modified Dulbecco medium (IMDM). After centrifugation at 150 × *g* for 5 min, the cell pellet was resuspended in IMDM, layered onto Percol separation solution (density 1.073 g/mL, Sigma‐Aldrich, St. Louis, MO) and centrifuged at 400 × *g* for 30 min. The cotton‐like cells at the interface were collected, rinsed and cultured with IMDM supplemented with 20% foetal bovine serum and 1% streptomycin/penicillin (Sigma‐Aldrich). The medium was changed after 24 h, and cells were maintained until approximately 80% confluence. After trypsinization, replating and cultivation until the second passage, plastic‐adherent, fibroblast‐like cells were considered BMSCs. To verify the multidirectional differentiation potential of BMSCs, we induced the cells into osteoblastic, adipogenic and chondrogenic cells for 20 to 30 days. After the differentiation of the cells to a certain extent, they were treated with the corresponding stains and finally photographed with a microscope (Olympus, Japan). These cells were harvested for characterization. Before use, the harvested BMSCs were stained and verified with fluorescein isothiocyanate‐conjugated mouse anti‐rat CD44, CD34, CD105 and CD45 (BD Bioscience, Franklin Lakes, NJ).

### 2.3 Exosome extraction and identification

The BMSCs were verified by their capability of osteogenic and adipogenic differentiation, as well as the expression of surface molecular markers of CD44, CD105, CD34 and CD45 examined by the flow cytometry. BMSCs were cultured in a serum‐free DMEM medium for 48 h at 37°C with 5% CO_2_. Recombinant human TNF‐α was obtained from Biolegend (San Diego, CA). BMSCs at the third passage were cultured with or without 20 ng/mL TNF‐α for 48 h. The resulting supernatant was gathered and underwent a series of procedures, including centrifugation (300 *g*, 4°C, 10 min), subsequent centrifugation (16,500×*g*, 4°C, 20 min) and filtration using 0.22 μm filters for sterilization. Following this, the supernatant experienced ultra‐high‐speed centrifugation (120,000×*g*, 70 min, 4°C) to yield precipitates, which were then resuspended in phosphate‐buffered saline (PBS) to form an exosomal suspension. Nanoparticle tracking analysis using NanoSight NS300 equipment (Malvern Instruments Ltd., Malvern, UK) was employed to disclose the size distribution of exosomes.

### 2.4 PFD rat model

The rat model of vaginal distention, previously documented,^19^, ^20^ was employed to simulate childbirth injury. In brief, a Foley balloon filled with 2.5–3.0 mL water was introduced into the rat vagina on Day 0, connected to a pressure transducer to exert pressure (approximately 0.15 kg) on the pelvic floor tissue. After 4 h, the Foley balloon was deflated and removed. Fourteen days post‐vaginal distention, the experimental rats were categorized into four groups: normal control, pelvic floor dysfunction (PFD), PFD rats treated with exosomes from BMSCs and PFD rats treated with exosomes (Exo) and TNF‐Exo. Specifically, exosomes (500 μL PBS) were administered via the caudal vein for seven consecutive days starting on Day 14. On Day 28, bladder baseline pressure, peak bladder pressure, leak point pressure (LPP) and void volume were measured to assess the severity of PFD.

### 2.5 Enzyme‐linked immunosorbent assay (ELISA)

The quantification of rat IL‐6, TNF‐α and MMP2 involved the use of ELISA kits (Abbexa, Cambridge, UK) following the provided manufacturer's guidelines. In summary, the specific primary antibody captured IL‐6, TNF‐α and MMP2 in the samples, which were subsequently identified using a biotin‐labelled secondary antibody. The assays were developed with avidin‐peroxidase and its substrate, and the readings were taken at 450 nm using a microplate reader.

### 2.6 Western blot

The anterior vaginal walls or exosomes were subjected to lysis, and the resulting soluble supernatants (30 μg) were separated through 12% sodium dodecyl sulfate–polyacrylamide gel electrophoresis and subsequently transferred onto polyvinylidene fluoride (PVDF) membranes. Following blocking with 5% nonfat milk, these PVDF membranes underwent incubation with primary antibodies targeting Elastin, Collagen I, Collagen III and β‐Actin (Santa Cruz Biotechnology Inc., Dallas, TX). Subsequently, peroxidase‐conjugated secondary antibodies (Sigma‐Aldrich) were applied, and the membranes were developed using Super Ecl Solution (GE Healthcare, Little Chalfont, Buckinghamshire, UK). β‐Actin served as a normalization reference for relative expression, and NIH‐Image J was utilized for analysis.

### 2.7 RT‐qPCR

Total RNA was isolated from the anterior vaginal walls and BMSCs using TRIzol reagent (Invitrogen) following the manufacturer's instructions. Subsequently, cDNA was synthesized in a reverse transcription process using a High‐Capacity cDNA Reverse Transcription Kit (Bio‐Rad, Hercules, CA) and a One Step PrimeScript miRNA cDNA Synthesis Kit (Takara, Dalian, China). The resulting cDNA was then amplified using SYBR Green master mix (Roche, Penzberg, Upper Bavaria, Germany). The amplification protocol involved an initial step at 95°C for 10 min, followed by 40 cycles of 95°C for 15 s and 60°C for 1 min on an ABI 7500 real‐time PCR system (Applied Biosystems, Foster City, CA). Expression data were normalized to GAPDH expression and quantified using the comparative ΔCT method. The primer sequences are provided as following: MMP2: forward 5′‐ CCTGTCTCCTGCTCTGTAG−3′, reverse 5′‐AGTAGCACCTGGGAGGGATA‐3′; Elastin, forward 5′‐CTTCCTGGTGGAGTTCCCGGTGGA‐3′, reverse 5′‐CCGATGCCACCAATACCACCGACA‐3′; COL1A1, forward 5′‐ATCAGCCCAAACCCCAAGGAGA‐3′, reverse 5′‐CGCAGGAAGGTCAGCTGGATAG‐3′; COL3A1, forward 5′‐TGATGGGATCCAATGAGGGAGA‐3′, reverse 5′‐GAGTCTCATGGCCTTGCGTGTTT‐3′; IL‐6, forward 5′‐GACTGATGTTGACAGCCACTGC‐3′, reverse 5′‐AGCCACTCCTTCTGTGACTCTAACT‐3′; TNF‐α, forward 5′‐CATGATCCGAGATGTGGAACTGGC‐3′, reverse 5′‐CTGGCTCAGCCACTCCAGC‐3′; GAPDH: forward 5′‐GACATGCCGCCTGGAGAAAC‐3′, reverse 5′‐ GACATGCCGCCTGGAGAAAC‐3′.

### 2.8 Statistical analysis

Statistical analyses were conducted utilizing SPSS software (SPSS Inc., Chicago, IL). The results are presented as the mean ± standard deviation (SD). Brown–Forsythe ANOVA test followed by Dunnett's T3 multiple comparisons test was employed for the comparison between groups, and statistical significance was determined at a *p* < 0.05.

## 3 RESULTS

### 3.1 IL‐6, TNF‐α and MMP2 were elevated in vaginal wall tissues from patients diagnosed with POP

**FIGURE 1:**
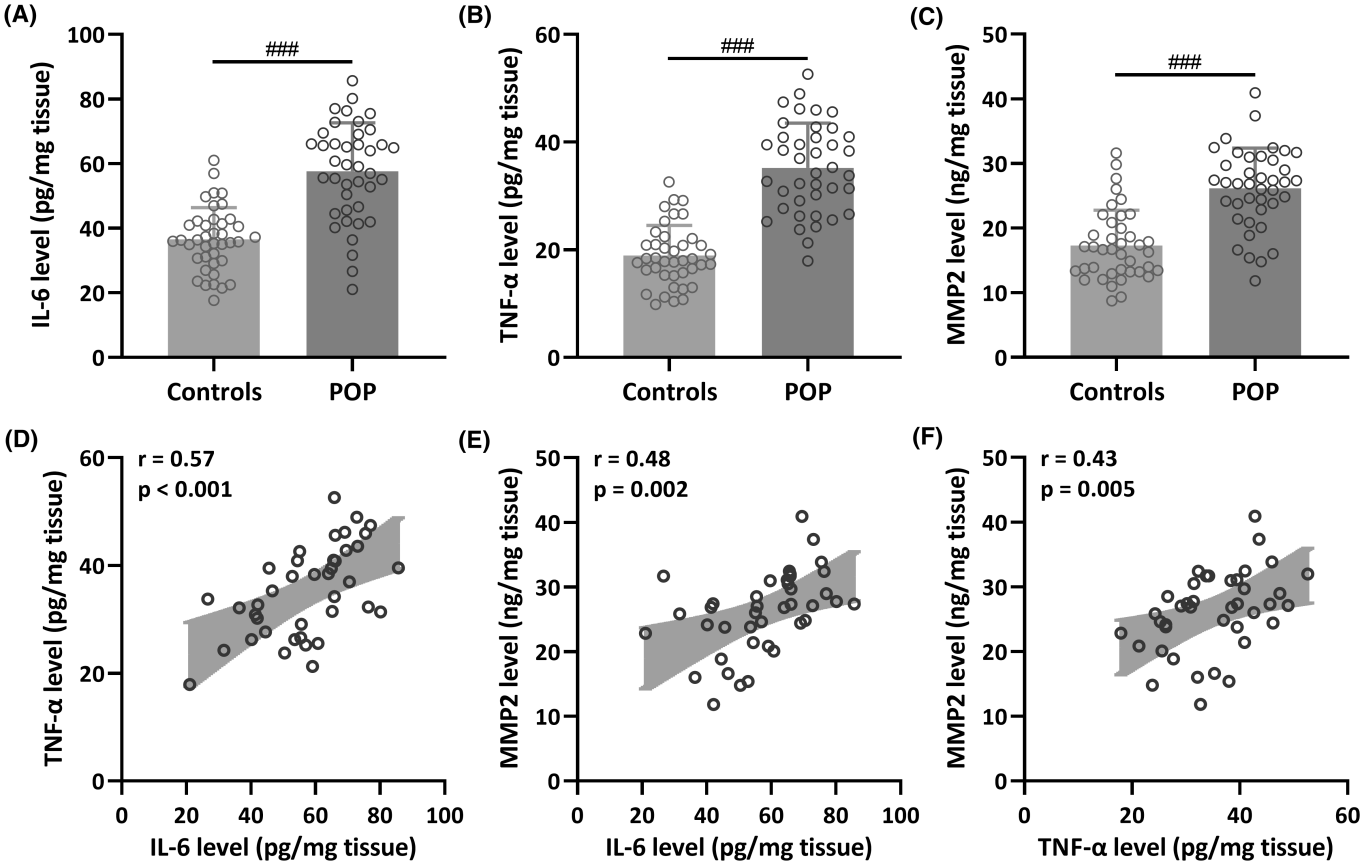
Comparisons of IL‐6 (A), TNF‐α (B) and MMP2 (C) levels in vaginal wall tissues between patients with pelvic organ prolapse (POP, *n* = 40) and controls (*n* = 40). Data were presented as mean ± SD. ###*p* < 0.001 from Unpaired *t*‐test with Welch's correction. Pearson correlation analysis of levels of IL‐6 with TNF‐α (D), IL‐6 with MMP2 (E) and TNF‐α with MMP2 (F) in vaginal wall tissues from patients with pelvic organ prolapse (POP, *n* = 40).

To compare the levels of IL‐6, TNF‐α and MMP2 in vaginal wall tissues between patients diagnosed with pelvic organ prolapse and a control group, we gathered a total of 80 vaginal wall tissue samples. This comprised 40 samples from patients with POP and 40 samples from control patients without POP. The outcomes indicated a significant increase in IL‐6 (Controls: 36.54 ± 9.84 pg/mg tissue; POP: 57.62 ± 15.01 pg/mg tissue; *p* < 0.001), TNF‐α (Controls: 18.92 ± 5.58 pg/mg tissue; POP: 35.16 ± 8.35 pg/mg tissue; *p* < 0.001) and MMP2 levels (Controls: 17.31 ± 5.44 pg/mg tissue; POP: 26.15 ± 6.20 pg/mg tissue; *p* < 0.001) in the vaginal wall tissues of patients with POP when compared to the control group (Figure 1A–C). Moreover, the results of Pearson correlation analysis demonstrated a robust positive correlation between IL‐6 level and TNF‐α level (*r* = 0.57, *p* < 0.001) (Figure 1D), IL‐6 level and MMP2 level (*r* = 0.48, *p* = 0.002) (Figure 1E), as well as TNF‐α level and MMP2 level (*r* = 0.43, *p* = 0.005) (Figure 1F).

### 3.2 TNF‐Exo successfully restored both void volume and bladder void pressure in PFD rats

**FIGURE 2:**
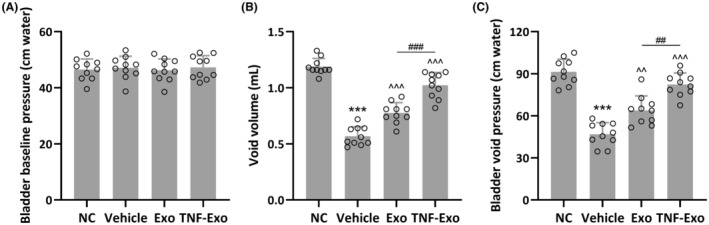
Exosomes isolated from cytokines treated BMSCs improved CMG in PFD rats. The rat PFD model was established and housed for 14 days, after which the rats were treated with exosomes daily for a further 7 days. CMG including bladder baseline pressure (A), void volume (B) and bladder void pressure (C) were then performed on all rats (*n* = 10 for each experimental group). Data were presented as mean ± SD. ***p < 0.001 compared with NC. ^^*p* < 0.01, ^^^*p* < 0.001 compared with PFD, ##*p* < 0.01, ###*p* < 0.001, between the two groups. Brown–Forsythe ANOVA test followed by Dunnett's T3 multiple comparisons test.

To investigate the biological effects of Exo and TNF‐Exo on rats with PFD, a rat PFD model was established. It is worth noting that TNF‐Exo treatment did not induce any obvious side‐effects in the normal healthy rats, including weight loss and abnormal behaviours (data not shown). As depicted in Figure 2A–C, the baseline pressure of the bladder showed no significant differences across all experimental groups (NC: 46.53 ± 3.74 cm water; Vehicle: 47.13 ± 4.09 cm water; Exo: 46.26 ± 4.02 cm water; TNF‐Exo: 47.28 ± 4.14 cm water; all *p* > 0.05) (Figure 2A). In comparison with normal control rats, those with PFD displayed a notable decrease in void volume (NC: 1.19 ± 0.07 mL; Vehicle: 0.57 ± 0.08 mL; Exo: 0.78 ± 0.09 mL; TNF‐Exo: 1.02 ± 0.11 mL; both *p* < 0.001 for Exo and TNF‐Exo groups vs. Vehicle group) (Figure 2B) and bladder void pressure (NC: 91.35 ± 9.14 cm water; Vehicle: 46.85 ± 8.25 cm water; Exo: 63.84 ± 10.35 cm water; TNF‐Exo: 82.35 ± 8.20 cm water; *p* = 0.0048 for Exo vs. Vehicle, and *p* < 0.001 for TNF‐Exo vs. Vehicle) (Figure 2C), indicating the successful construction of the rat PFD model. Following daily treatment with exosomes for 7 days, both the Exo and TNF‐Exo groups exhibited increased void volume and bladder void pressure. Notably, TNF‐Exo treatment demonstrated more effective restoration of void volume and bladder void pressure compared with Exo treatment (*p* < 0.001 for void volume and *p* = 0.0021 for bladder void pressure).

### 3.3 TNF‐Exo effectively improved peak bladder pressure and leak point pressure in PFD rats

**FIGURE 3:**
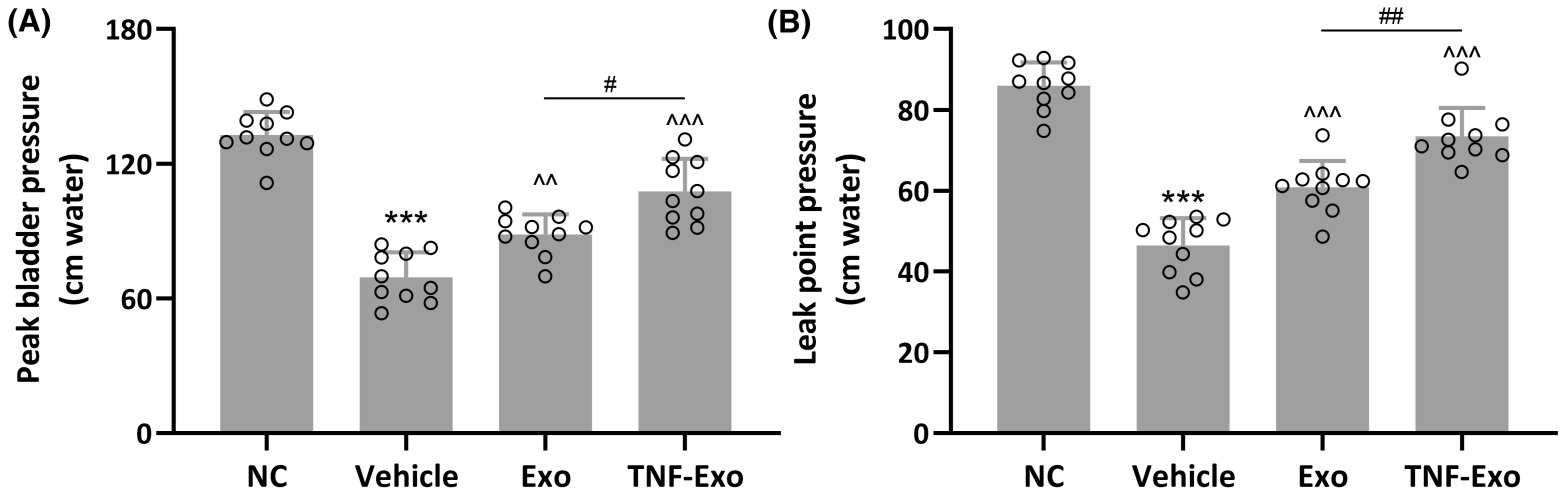
Exosomes isolated from cytokines treated BMSCs improved LPP in PFD rats. The rat PFD model was established and housed for 14 days, after which the rats were treated with exosomes daily for a further 7 days. Peak bladder pressure (A) and leak point pressure (B) were then performed on all rats (*n* = 10 for each experimental group). Data were presented as mean ± SD. ***p < 0.001 compared with NC. ^^*p* < 0.01, ^^^*p* < 0.001 compared with PFD, #*p* < 0.05, ##*p* < 0.01, between the two groups. Brown–Forsythe ANOVA test followed by Dunnett's T3 multiple comparisons test.

PFD rats exhibited a noteworthy reduction in peak bladder pressure (NC: 132.80 ± 10.24 cm water; Vehicle: 69.45 ± 11.03 cm water; *p* < 0.001) and leak point pressure (NC: 85.98 ± 5.79 cm water; Vehicle: 46.47 ± 6.76 cm water; *p* < 0.001) compared with control rats (Figure 3A,B). As anticipated, the administration of Exo partially reinstated the diminished peak bladder pressure (Exo: 88.45 ± 8.97 cm water; *p* = 0.0033 vs. Vehicle) and leak point pressure (Exo: 60.88 ± 6.49 cm water; *p* < 0.001 vs. Vehicle) observed in PFD rats (Figure 3A,B). Moreover, the injection of TNF‐Exo further reversed the decreased peak bladder pressure (TNF‐Exo: 107.70 ± 14.43 cm water; *p* < 0.001 vs. Exo) and leak point pressure (TNF‐Exo: 73.47 ± 7.00 cm water; *p* < 0.001 vs. Exo) (Figure 3A,B).

### 3.4 TNF‐Exo enhanced the expression levels of Elastin, Collagen I and Collagen III in the anterior vaginal walls of PFD rats

**FIGURE 4:**
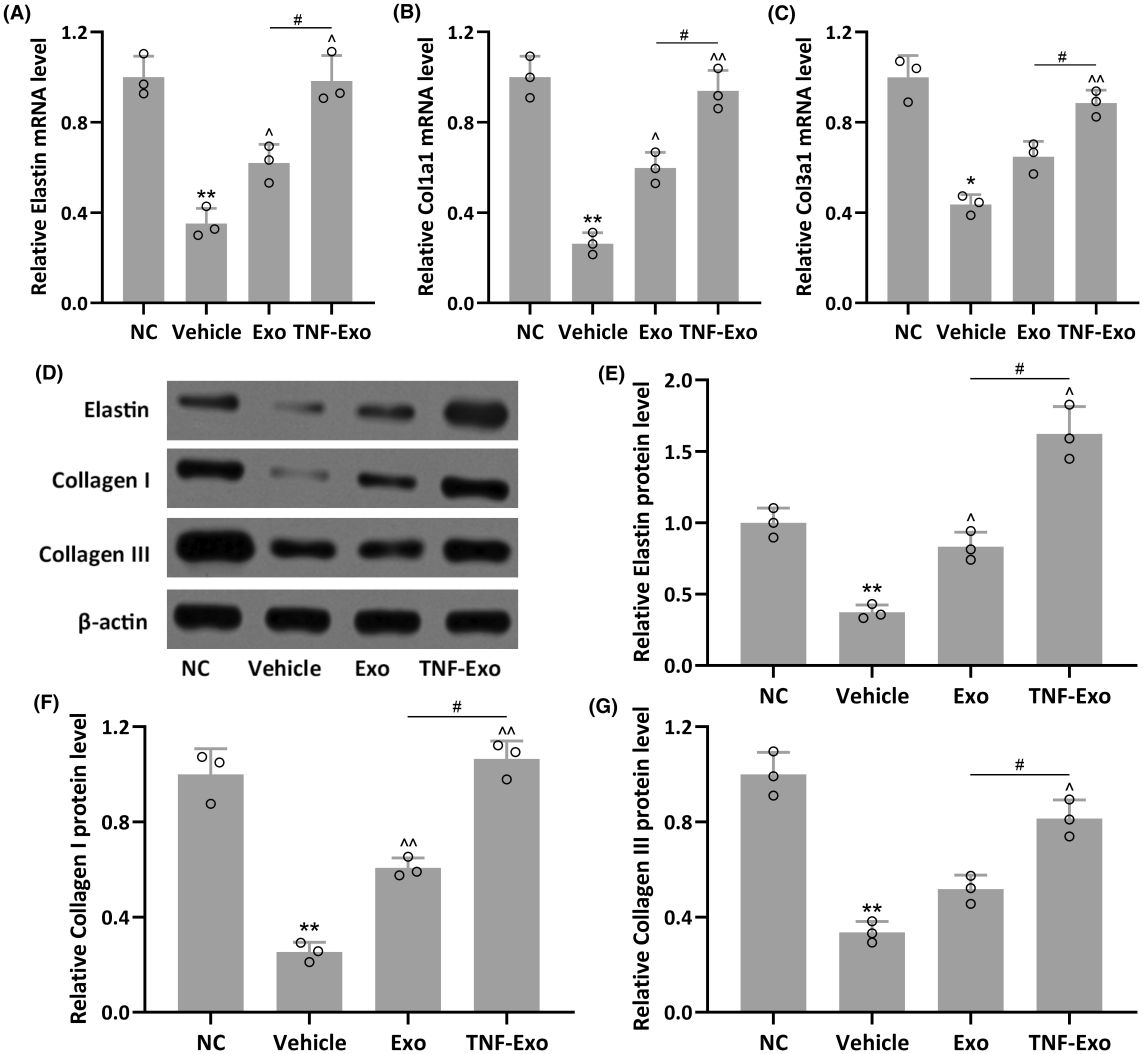
Exosomes isolated from cytokines treated BMSCs improved the expressions of Elastin, Collagen I and Collagen III in PFD rats. The rat PFD model was established and housed for 14 days, after which the rats were treated with exosomes daily for a further 7 days. The anterior vaginal walls were collected, and qRT‐PCR was used to measure the mRNA expressions of Elastin (A), Col1a1 (B) and Col3a1 (C) in the anterior vaginal walls. Western blotting was used to measure the protein expressions of Elastin, Collagen I and Collagen III in the anterior vaginal walls (D) β‐Actin was used as a loading control, and the expressions were normalized to NC (E–G). Data were presented as mean ± SD. *p < 0.05, **p < 0.01, compared with NC. ^*p* < 0.05, ^^*p* < 0.01 compared with PFD, #*p* < 0.05 between the two groups. Brown–Forsythe ANOVA test followed by Dunnett's T3 multiple comparisons test.

**FIGURE 5:**
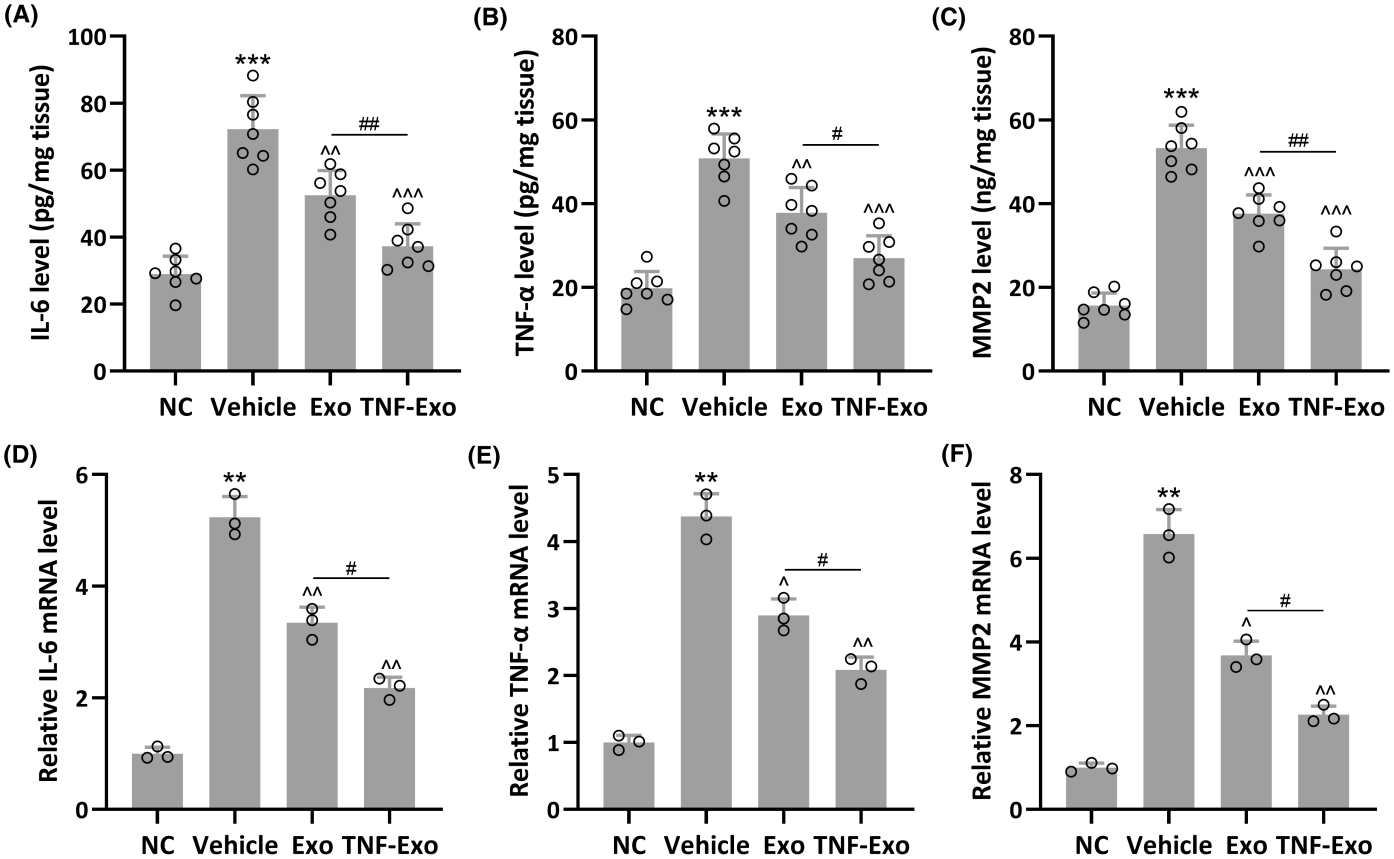
Exosomes isolated from cytokines treated BMSCs inhibited IL‐6, TNF‐α and MMP2 in PFD rats. The rat PFD model was established and housed for 14 days, after which the rats were treated with exosomes daily for a further 7 days. The anterior vaginal walls were collected, and the levels of IL‐6 (A), TNF‐α (B) and MMP2 (C) were measured by ELISA. qRT‐PCR was used to measure the mRNA expressions of IL‐6 (D), TNF‐α (E) and MMP2 (F) in the anterior vaginal walls. Data were presented as mean ± SD. **p < 0.01, ***p < 0.001, compared with NC. ^*p* < 0.05, ^^*p* < 0.01, ^^^*p* < 0.001 compared with PFD, #*p* < 0.05, ##*p* < 0.01 between the two groups. Brown–Forsythe ANOVA test followed by Dunnett's T3 multiple comparisons test.

The mRNA and protein levels of Elastin, Collagen I and Collagen III in the anterior vaginal walls of PFD rats were further assessed. Reduced mRNA levels of Elastin, Col1a1 and Col3a1 were observed in PFD rats when compared to NC group (*p* = 0.0026 for Elastin; *p* = 0.0044 for Col1a1; *p* = 0.0101 for Col3a1) (Figures 4A and 5C). Exo, especially TNF‐Exo, treatment significantly enhanced their mRNA levels in PFD rats. Similarly, Exo treatment partially restored the expression of Elastin, Collagen I and Collagen III, while TNF‐Exo treatment nearly completely restored their protein expression when compared to the NC group (Figures 4D and 5G).

### 3.5 TNF‐Exo suppressed the expression levels of IL‐6, TNF‐α and MMP2 in the anterior vaginal walls of PFD rats

As anticipated, elevated levels of IL‐6 (NC: 28.97 ± 5.34 pg/mg tissue; Vehicle: 72.26 ± 10.01 pg/mg tissue), TNF‐α (NC: 19.83 ± 4.01 pg/mg tissue; Vehicle: 50.82 ± 5.85 pg/mg tissue) and MMP2 production (NC: 15.66 ± 3.02 pg/mg tissue; Vehicle: 53.26 ± 5.51 pg/mg tissue) were observed in the anterior vaginal walls of PFD rats (Figure 5A–C). Exo treatment partially restored the production of IL‐6 (52.53 ± 7.39 pg/mg tissue), TNF‐α (37.82 ± 6.03 pg/mg tissue) and MMP2 (37.65 ± 4.47 pg/mg tissue), whereas TNF‐Exo treatment almost completely restored IL‐6 (37.32 ± 6.63 pg/mg tissue), TNF‐α (26.97 ± 5.34 pg/mg tissue) and MMP3 (24.32 ± 5.02 pg/mg tissue) production. Similar trends were found in mRNA expression of IL‐6, TNF‐α and MMP2 in these groups (Figure 5D–F). It was observed that TNF‐Exo treatment could greatly enhance the mRNA expression of IL‐6, TNF‐α and MMP2 in PFD rats.

## 4 DISCUSSION

The weakening of pelvic connective tissues is considered a contributing factor to PFD. Elastin plays a crucial role in the composition of vaginal and pelvic floor connective tissues.^21^ Increasing attention in research has been directed towards understanding the role of elastin in the pathophysiological development of PFD, particularly in stress urinary incontinence and POP, both of which are linked to injuries sustained during childbirth.^22^ The stretching of the vagina during delivery leads to the degradation of elastin fibres. The resulting disorganized elastin fibres contribute to dysfunction, ultimately causing tissue stiffness.^23^, ^24^ The prevalence of PFD is high among women, and current treatment options are considered suboptimal. BMSCs show significant potential in the treatment of various diseases, including the reconstruction of connective tissues.^25^ Several studies have indicated that both MSCs and MSC‐derived extracellular vesicles (EVs) exhibit similar therapeutic effects on specific diseases, suggesting that MSCs may exert their therapeutic effects by secreting EVs.^26^, ^27^ Small EVs, in particular, offer advantages such as their diminutive size, more direct action, superior therapeutic effects and the ability to navigate through the bloodstream without getting trapped in capillaries.^28^, ^29^

Moreover, earlier research has noted that EVs released by MSCs derived from the umbilical cord and bone marrow do not induce rejection in mice.^30^ In our recent study, we demonstrated that exosomes derived from genetically engineered BMSCs with miR‐195‐5p can target the TGF‐β /Smad7 pathway.^10^ This targeting action facilitates the repair of connective tissues and restoration of damaged urodynamic function in PFD rats. Interestingly, our current study further showed that exosomes derived from TNF‐α‐treated BMSCs alleviate pelvic floor dysfunction in rats to restore the function of virginal distention, with more treatment benefit than exosomes derived from BMSCs. TNF‐Exo injection exhibited superior improvement effects on conscious cystometry (CMG) and LPP in PFD rats.

The potential molecular mechanism underlying this phenomenon could be linked to the expression of Elastin, Collagen I and Collagen III in the anterior vaginal walls of PFD rats. Elastin, as an insoluble polymer of tropoelastin that forms elastic fibres in the extracellular matrix (ECM), has the ability to promote tissue regeneration and return to its original shape without requiring additional energy. Women with PFD typically exhibit increased elastin degradation and abnormal elastin synthesis. Our prior research has shown that BMSCs expressing elastin play a beneficial role in the repair of pelvic floor tissues. Transfection with microRNA‐29a‐3p induces elastin expression in BMSCs, thereby alleviating PFD through new tissue growth and collagen deposition.

Collagen I and Collagen III are predominant components of the extracellular matrix, significantly contributing to the biomechanical properties of the vagina.^31^ These two types of collagen have distinct fibre diameters, resulting in variations in their mechanical properties. Collagen I imparts tensile strength and stiffness, while Collagen III prevails in more flexible tissue.^32^, ^33^ Our observations indicate that TNF‐Exo treatment induces an elevated expression of Elastin, Collagen I and Collagen III in the anterior vaginal walls of PFD rats. This elevation contributes to the restoration of the biological function of the vagina in PFD rats.

There are three crucial pro‐inflammatory cytokines implicated in adhesion formation/reformation: IL‐1 and TNF‐α, both playing key roles in the initial phase of wound healing, are produced by activated macrophages in peritoneal fluid, while IL‐6 is expressed by macrophages and its production is upregulated by IL‐1 during the inflammatory process.^34^, ^35^ IL‐1 and TNF‐α are potent inducers of IL‐6. These cytokines are deemed significant as they extensively interact with the fibrinolytic pathway and contribute directly or indirectly to the remodelling of the extracellular matrix.^36^, ^37^ In our study, we observed that treatment with TNF‐Exo suppressed the elevated levels of IL‐6, TNF‐α and MMP2 in the anterior vaginal walls of PFD rats, potentially alleviating PFD symptoms. The precise underlying mechanism remains unknown. One plausible explanation is that the preconditioning of BMSCs with TNF‐α was employed to simulate the inflammatory environment in the gut, compelling BMSCs to secrete substances inhibiting TNF‐α. Previous research involving the pretreatment of BMSCs with TNF‐α and IFN‐γ demonstrated enhanced immunomodulatory capacity in BMSCs‐derived EVs, as verified through sequencing and cellular experiments.^38^, ^39^

It is important to note some limitations. The mechanism by which TNF‐Exo regulates the expression of multiple proteins (Elastin, Collagen I, Collagen III, IL‐6, TNF‐α and MMP2) in the anterior vaginal walls of PFD rats remains not fully understood. Additionally, the differential protein profile of Exo and TNF‐Exo was not investigated. Our future studies will aim to address these critical questions.

## 5 CONCLUSION

Exosomes derived from TNF‐α treated BMSCs demonstrate the ability to alleviate pelvic floor dysfunction in rats. The genetic engineering of BMSCs can alter the content in the exosomes, presenting a novel therapeutical method for treating PFD.

## AUTHOR CONTRIBUTIONS

**Chenchen Zhou:** Data curation (lead); validation (lead); writing – original draft (lead); writing – review and editing (lead). **Yuelin Wu:** Data curation (supporting); validation (supporting); writing – original draft (supporting); writing – review and editing (supporting). **Sheng Wan:** Data curation (supporting); validation (supporting); writing – original draft (supporting); writing – review and editing (supporting). **Liqun Lou:** Data curation (supporting); supervision (supporting); validation (supporting); writing – original draft (supporting); writing – review and editing (supporting). **Shengyi Gu:** Data curation (supporting); validation (supporting); writing – original draft (supporting); writing – review and editing (supporting). **Jing Peng:** Data curation (supporting); validation (supporting); writing – original draft (supporting); writing – review and editing (supporting). **Shifeng Zhao:** Data curation (supporting); validation (supporting); writing – original draft (supporting); writing – review and editing (supporting). **Xiaolin Hua:** Data curation (lead); funding acquisition (lead); resources (lead); supervision (lead); validation (lead); writing – original draft (lead); writing – review and editing (lead).

## FUNDING INFORMATION

The study was supported National Natural Science Foundation of China (82071629), Shanghai Science and Technology Innovation Fund (23Y11909400) and Research Fund from Shanghai Municipal Health Commission (2022XD004).

## CONFLICT OF INTEREST STATEMENT

The authors declare that they have no competing interests.

## Data Availability

The raw data supporting the conclusions of this article will be made available by the authors, without undue reservation.
